# A sensitive method for the quantification of virion-sense and complementary-sense DNA strands of circular single-stranded DNA viruses

**DOI:** 10.1038/srep06438

**Published:** 2014-09-22

**Authors:** Edgar A. Rodríguez-Negrete, Sonia Sánchez-Campos, M. Carmen Cañizares, Jesús Navas-Castillo, Enrique Moriones, Eduardo R. Bejarano, Ana Grande-Pérez

**Affiliations:** 1 Instituto de Hortofruticultura Subtropical y Mediterránea “La Mayora”, Universidad de Málaga - Consejo Superior de Investigaciones Científicas, (IHSM-UMA-CSIC) Área de Genética, Campus de Teatinos, 29071 Málaga, Spain; 2 Instituto de Hortofruticultura Subtropical y Mediterránea “La Mayora”, Universidad de Málaga - Consejo Superior de Investigaciones Científicas, (IHSM-UMA-CSIC) Estación Experimental “La Mayora”, 29750 Algarrobo-Costa, Málaga, Spain; 3 Current address: Centro de Investigación y Estudios Avanzados (Cinvestav) Irapuato Km. 9.6 Libramiento Norte Irapuato, Guanajuato. 36821 Mexico.

## Abstract

Circular single-stranded DNA (ssDNA) viruses are the smallest viruses known to infect eukaryotes. High recombination and mutation rates have conferred these viruses with an evolutionary potential that has facilitated their emergence. Their damaging effects on livestock (circoviruses) and crops (geminiviruses and nanoviruses), and the ubiquity of anelloviruses in human populations and other mammalian species, have resulted in increased interest in better understanding their epidemiology and infection mechanisms. Circular ssDNA viral replication involves the synthesis of dsDNA intermediates containing complementary-sense (CS) and virion-sense (VS) strands. Precise quantification of VS and CS accumulation during viral infections can provide insights into the molecular mechanisms underlying viral replication and the host invasion process. Although qPCR protocols for quantifying viral molecules exist, none of them discriminate VS and CS strands. Here, using a two-step qPCR protocol we have quantified VS and CS molecule accumulation during the infection process of *Tomato yellow leaf curl virus* (TYLCV) and *Tomato yellow leaf curl Sardinia virus* (TYLCSV) (genus *Begomovirus*, family *Geminiviridae*). Our results show that the VS/CS strand ratio and overall dsDNA amounts vary throughout the infection process. Moreover, we show that these values depend on the virus-host combination, and that most CS strands are present as double-stranded molecules.

Eukaryotic circular single-stranded DNA (ssDNA) viruses are currently grouped into four families according to their morphology, genome organization and host range: *Anelloviridae*, *Circoviridae*, *Geminiviridae* and *Nanoviridae*^1^. Despite having a small size and limited protein coding capacity, their rapid evolution rate, enormous diversity and prevalence in numerous environments have resulted in circular ssDNA viruses emerging as an important class of pathogen^2^. Interest in these viruses has increased as their harmful effects on livestock (circoviruses) and crops (geminiviruses and nanoviruses) have emerged over recent decades. Moreover, it has now been discovered that anelloviruses are present in humans and other mammalian species, and whose plasma viral load increases substantially during immunosuppressive therapy^3^,^4^.

The circular ssDNA genomes of geminiviruses, nanoviruses and most circoviruses, encode a conserved rolling circle replication protein (Rep) and contain a stem loop structure at the origin of replication^2^. They are believed to replicate by rolling circle replication (RCR) via the formation of a circular dsDNA intermediate^5^,^6^. Although less studied than in Rep-encoding viruses, the replication of anelloviruses is also suspected to occur via RCR since the intergenic region of their genome contains potential stem-loop structures and circular dsDNA forms have been found in infected tissues^4^,^7^.

Most experimental data on the circular replication mechanism of ssDNA viruses have been obtained from geminiviruses^8^. Geminiviruses are insect-transmitted viruses composed of a genome encapsulated by twinned quasi-icosahedral (geminate) virions^9^,^10^. Geminiviruses have monopartite or bipartite genomes, where each 2.5–3 kb genomic component encodes a maximum of six proteins distributed between both virion-sense (VS) and complementary-sense (CS) strands. ssDNA viral infection requires replication of the encapsidated VS strand and the expression of ORFs on both VS and CS strands in the case of geminiviruses and circoviruses. To replicate their genomes, circular VS ssDNA contained in the infectious viral particle must be transported to the nucleus where it is converted to dsDNA through an unknown mechanism that relies completely on host replication machinery. This dsDNA is then used to amplify the viral genome by RCR. In geminiviruses, additional replication mechanisms such as recombination dependent replication (RDR), and complementary-strand replication (CSR) are also involved in viral DNA replication^11^,^12^. Amplification involves the generation of additional dsDNA molecules that function as replication intermediates for the production of VS ssDNA, which is finally encapsidated to produce infectious viral particles. The fact that VS molecules are generated from CS templates and that VS-encoded proteins are involved in viral replication (Rep and C3), indicate that CS strand accumulation represents a marker for viral replication and expression in infected cells^12^,^13^,^14^,^15^.

Although the accumulation of viral ds and ssDNA molecules has been shown to occur during infection with circular ssDNA viruses^7^,^11^,^12^,^16^, quantitative data regarding the relative accumulation of VS and CS strands, or that of ssDNA versus dsDNA over the course of an infection are not available. Existing strand-specific PCR protocols developed for circular ssDNA viruses are not quantitative^7^,^17^ and established quantitative PCR (qPCR) protocols do not discriminate between the two viral strands^18^,^19^,^20^,^21^,^22^,^23^. We anticipate that quantitative determination of VS and CS strands during infection will provide important insights into circular ssDNA viral replication and infection dynamics. With this aim, we have developed a quantitative two-step qPCR protocol for the sensitive and reliable quantification of VS and CS molecules. This method was used to monitor infections of isolates of two monopartite begomoviruses (genus *Begomovirus*, family *Geminiviridae*): *Tomato yellow leaf curl virus* (TYLCV) and *Tomato yellow leaf curl Sardinia virus* (TYLCSV) in two host plant species: *Solanum lycopersicum* (tomato) and *Nicotiana benthamiana* (a close relative of tobacco). Both viruses are involved in the tomato yellow leaf curl disease, one of the most devastating viral diseases affecting tomato crops in warm areas worldwide^24^. Our results make it possible, for the first time, to precisely quantify VS and CS strand accumulation during the infection process. Using this method we show that VS/CS ratios vary as a function of infection stage, plant host and virus species. Moreover, our findings indicate that most CS molecules are present as part of dsDNA intermediates.

## Results

### A two-step qPCR technique for the quantification of VS and CS viral DNA molecules

**Figure 1:**
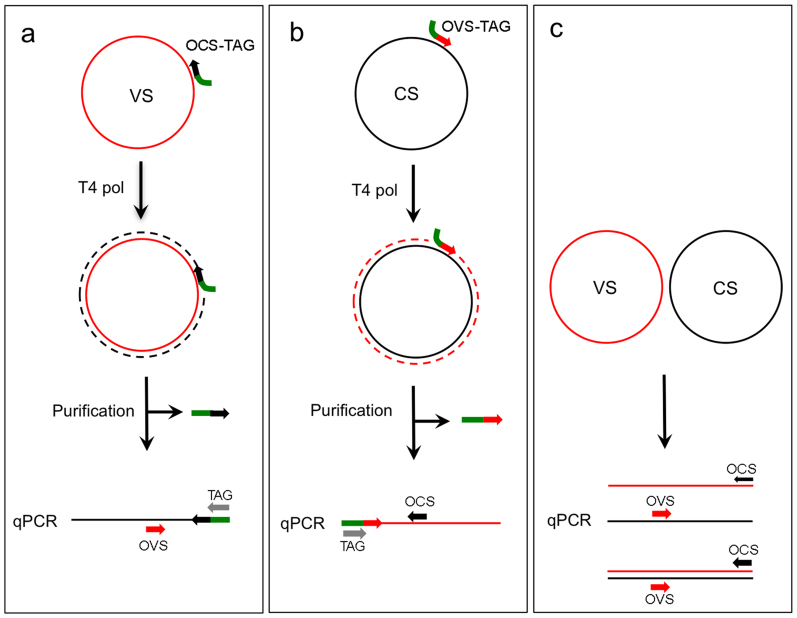
Schematic representation of a two-step quantitative PCR (qPCR) procedure for the quantification of virion-sense (VS) and complementary-sense (CS) DNA molecules. (A) Amplification of the VS strand using the OCS-TAG primer for T4 DNA polymerase extension and subsequent qPCR amplification with OVS and TAG primers. (B) Amplification of the CS strand using the OVS-TAG primer for T4 DNA polymerase extension followed by qPCR amplification with OCS and TAG primers. (C) qPCR to quantify both VS and CS strands using OVS and OCS primers. Primers used for T4 polymerase extension are removed prior to performing qPCR.

We have designed a simple and reliable two-step procedure for the quantification of VS and CS strand molecules present in a sample. The first step involves copying denatured viral strands using the T4 DNA polymerase with strand-specific primers (Figure 1 A and B). To this end, two oligonucleotide primers were designed with a 5′ virus-unrelated sequence (TAG) and a 3′ region complementary to either the virion-sense (OCS-TAG), or complementary-sense (OVS-TAG) strands (Figure 1A). Using one of these strand-specific oligonucleotides as primer, a single tagged copy of VS (using OCS-TAG) or CS (using OVS-TAG) strands is produced when T4 DNA polymerase reaches the 5′ end of the extended primer, as replication halts due to this enzyme's lack of strand-displacement activity. Next, unincorporated oligonucleotides are removed using a silica-membrane spin column, and the amount of purified DNA corresponding to VS or CS strands quantified by qPCR. Following this method an amplicon of 188 bp of the TYLCSV coat protein-coding region was amplified by qPCR using a TAG oligonucleotide, which is identical to the 5′ half of the extended primer, and a specific primer complementary to the T4-synthesized strand. Thus, to quantify VS strands, OCS-TAG was used for T4 primer extension followed by qPCR amplification with OVS and TAG primers (Figure 1A). Conversely, for CS strands, OVS-TAG was extended by T4 polymerase and OCS and TAG were used for the qPCR assay (Figure 1B). Total amount of viral DNA (VS + CS) present in the samples was quantified by standard qPCR using OVS and OCS primers (Figure 1C).

In order to evaluate method specificity, we generated circular single-stranded VS or CS TYLCSV DNA molecules. Full-length TYLCSV genomes cloned into pBSK phagemids (pBTSvs in virion-sense and pBTScs in complementary-sense) were used to produce circular ssDNA molecules harbouring VS (ssVS) or CS (ssCS) viral sequences. These ssDNAs were purified and employed as templates for T4 primer extension with either OCS-TAG or OVS-TAG followed by qPCR with TAG + OVS or TAG + OCS primer pairs, respectively. Post-amplification dissociation analysis showed single melting curves corresponding to individual bands when analyzed by gel electrophoresis (See Supplementary Fig. S1, A and B). A band of the expected size (188 bp) was detected when OCS-TAG was used as a primer for first strand synthesis of ssVS but not ssCS molecules (Supplementary Fig. S1A). Conversely, a similar band was observed when using OVS-TAG as a primer for ssCS but not ssVS first strand synthesis (Supplementary Fig. S1B). These results demonstrate that the two-step procedure can be used to detect VS and CS ssDNA circular templates in a specific manner.

### Sensitivity and analytical specificity of the absolute quantification of TYLCSV and TYLCV VS and CS strands

**Figure 2:**
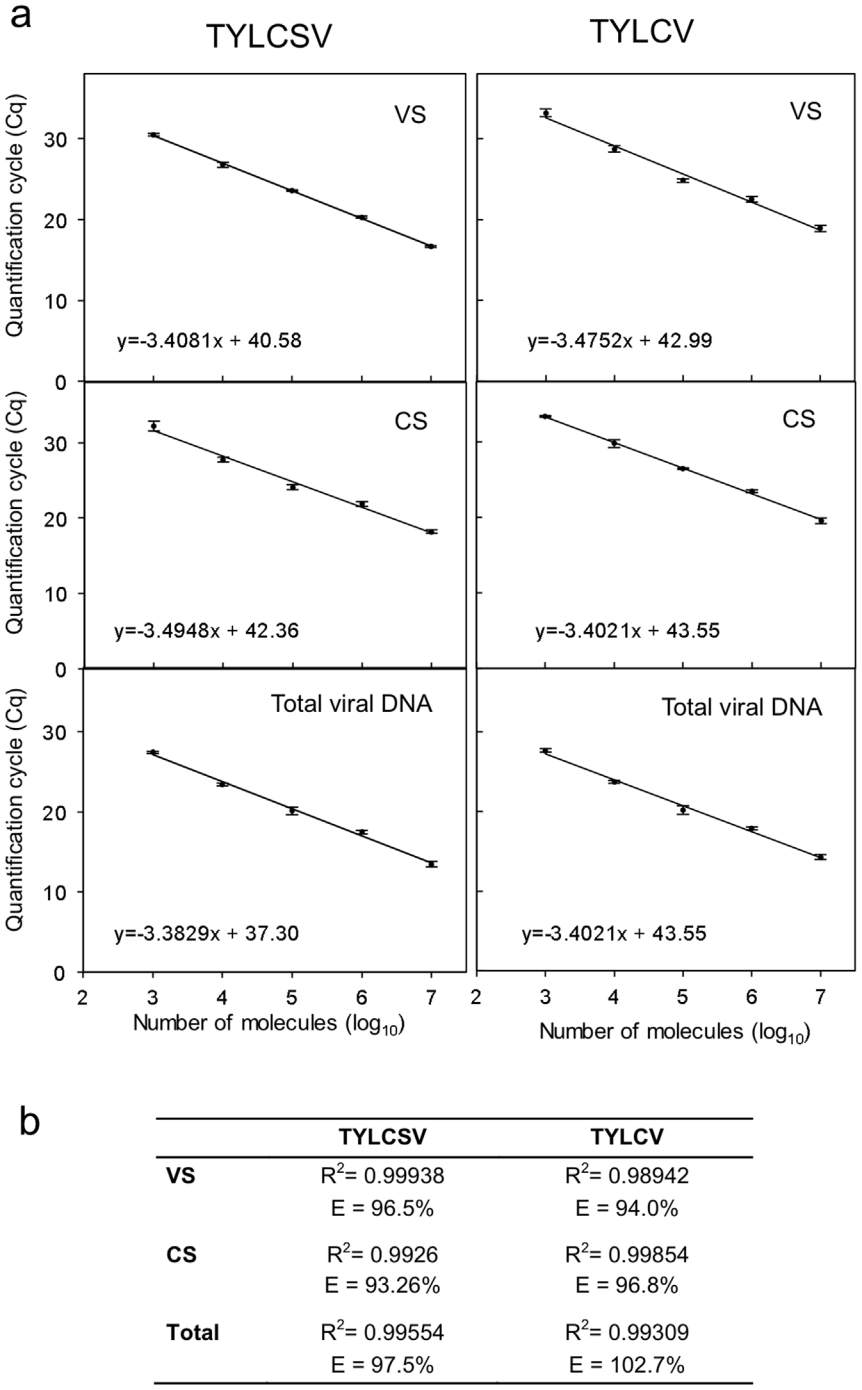
Quantification of virion-sense (VS) and complementary-sense (CS) DNA molecules of *Tomato yellow leaf curl Sardinia virus* (TYLCSV) and *Tomato yellow leaf curl virus* (TYLCV). (A) Standard curves for the quantification of VS, CS, and total viral DNA of TYLCSV and TYLCV are shown. Dilution series containing between 1 × 10^3^ and 1 × 10^7^ copies of circular ssDNA containing full-length TYLCSV or TYLCV genomes in the appropriate orientation (VS or CS), or dsDNA phagemids carrying a full-length copy of the viral genome (Total viral DNA) were used as quantification standards. Data represent the average of three independent qPCR replicates. Bars represent SD. Equations used for regression plot calculation are indicated in each case. (B) Regression plot correlation coefficients and qPCR efficiency values obtained.

**Figure 3:**
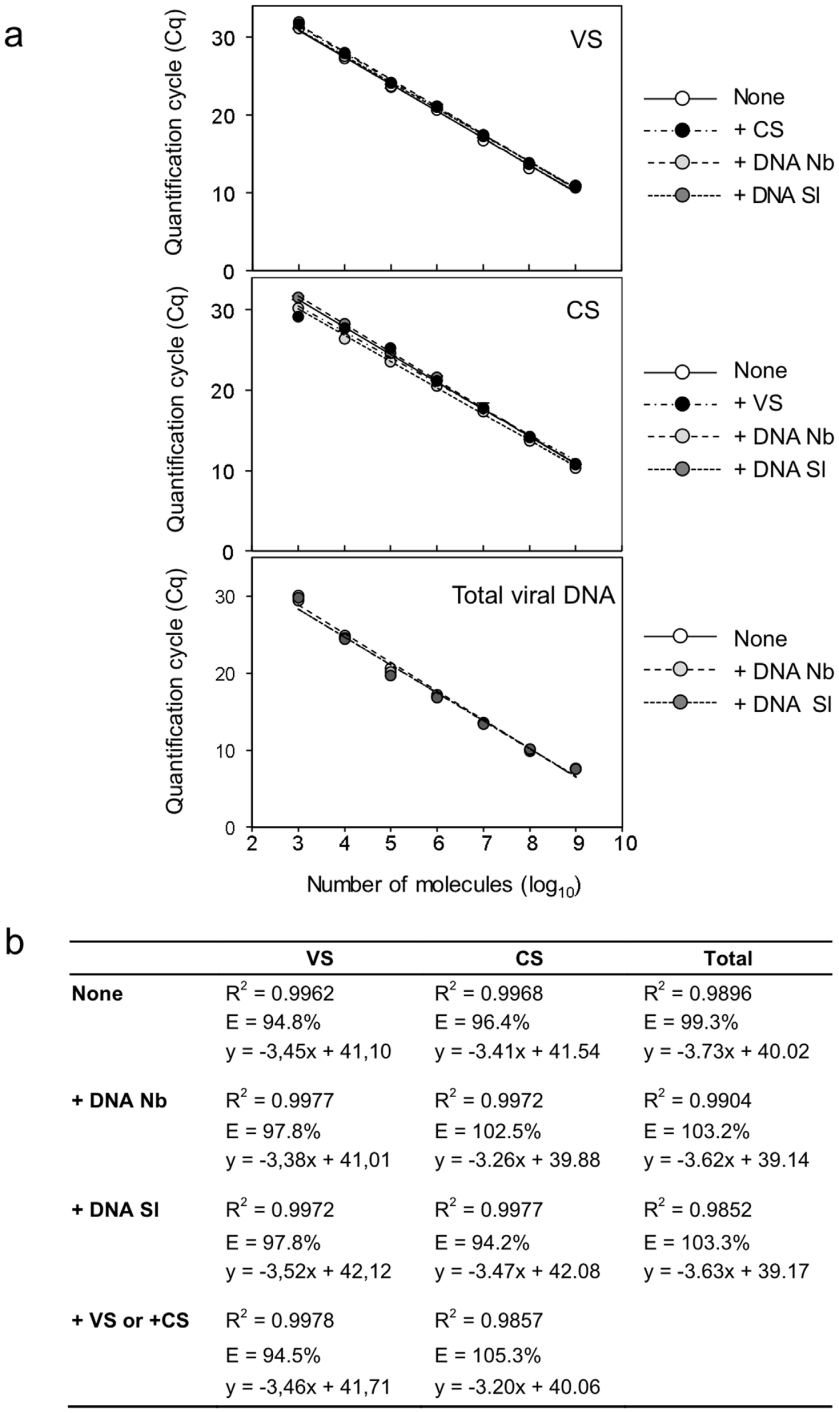
Standard curves for the absolute quantification of viral molecules. The relationship between quantification cycle (C_q_) and the amount of viral molecules used is shown (A). Dilution series containing between 1 × 10^3^ to 1 × 10^7^ copies of *Tomato yellow leaf curl Sardinia virus* (TYLCSV) virion-sense (VS) or complementary-sense (CS) circular ssDNA strands; or dsDNA phagemids carrying a full-length copy of the TYLCSV genome (Total viral DNA) were used for quantification. Amplification was performed according to the methods described in Figure 1. Prior to extension and amplification, four types of sample were prepared for each VS, CS and total viral DNA by adding water (negative control), an excess (10^8^ copies) of the corresponding complementary strand (+CS for VS determination and +VS for CS determination) or 200 ng of *Nicotiana benthamiana* (+DNA Nb) or *Solanum lycopersicum* (tomato) (+DNA Sl) genomic DNA. Data shown represent the average of two independent qPCR replicates. (B) Regression plot equations, correlation coefficients and qPCR efficiency values obtained.

Next, we tested the sensitivity and specificity of the two-step qPCR procedure to determine the number of TYLCSV and TYLCV DNA molecules of each polarity. Dilution series containing between 10^3^ and 10^7^ molecules of circular VS and CS ssDNA strands were prepared from phagemids containing full-length TYLCV and TYLCSV genomes in the appropriate orientation (see the Materials and Methods section for details). Similar dilution series were also prepared of dsDNA viral molecules using dsDNA phagemids. Using these test samples we quantified circular CS and VS ssDNA strands and total viral DNA following the procedure outlined in Figure 1. The quantification of both TYLCSV and TYLCV ssDNA was highly reproducible with low intra-assay quantification cycle (Cq) value variability (SD from 0.1 to 0.6) between standard curves of three independent PCR replicas. Data repeatability was accurate in the dynamic range of 10^7^ to 10^3^ molecules. However, higher C_q_ value variability was observed with samples containing 10^2^ molecules (SD ~ 2). Accordingly, the limit of detection (LOD) for the technique was fixed at 10^3^ molecules. On the other hand, inter-assay tests from three independent standard curves for each condition, summarised in Figure 2A, showed low C_q_ variation, high correlation coefficients and calculated PCR efficiencies ranging from 93.3% to 102.7% (Figure 2B) across the entire linear dynamic range assayed, indicating that the assay is highly reproducible. Importantly, PCR efficiency values and qPCR correlation coefficient regression plots remained unchanged when 10^8^ copies of ssCS or ssVS strands were added to the dilution series for quantification of TYLCSV VS or CS molecules (Figure 3, +CS and +VS, respectively). Similarly, the presence of 200 ng of *S. lycopersicum* or *N. benthamiana* genomic DNA did not interfere with the quantification of VS or CS strands (Figure 3, +DNA Sl and +DNA Nb, respectively), or the measurement of total DNA by standard qPCR (Figure 3A, Total viral DNA; Figure 3B, Total). These results indicate that our protocol is a robust and consistent assay even in the presence of large amounts of non-specific DNA.

**Figure 4:**
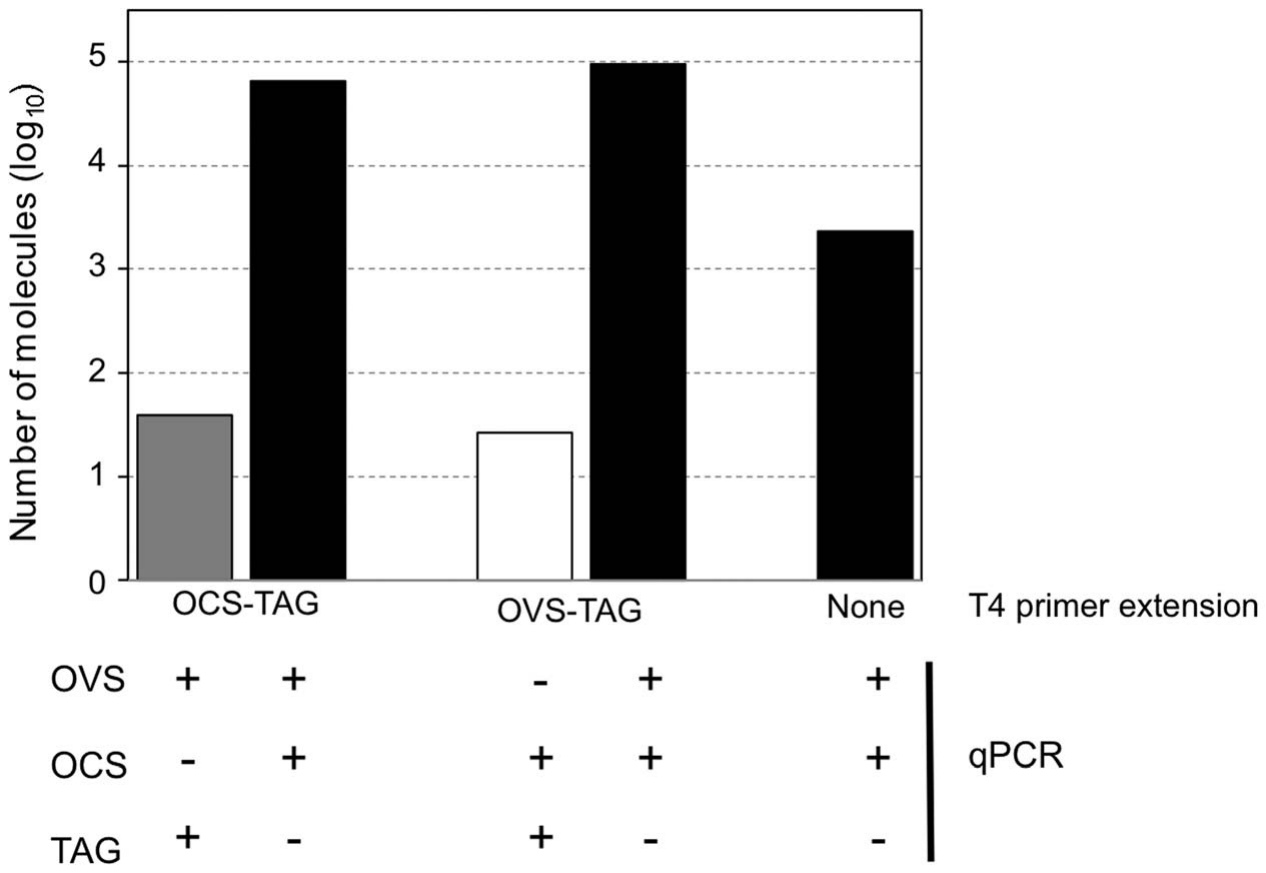
Absolute quantification of *Tomato yellow leaf curl Sardinia virus* (TYLCSV) virion-sense (VS) and complementary-sense (CS) ssDNA molecules, in the presence of an equimolar amount of dsDNA molecules of the same virus. Circular ssDNA molecules bearing the TYLCSV VS strand (10^6^ copies) were mixed with the same quantity of circular ssDNA bearing the TYLCSV CS strand and with 10^6^ molecules of phagemid dsDNA carrying the TYLCSV genome. The mix was denatured and used as template for T4 DNA polymerase primer extension with the primers described in Fig. 1, followed by qPCR with the indicated primer combinations for quantification of VS strands (grey bar), CS strands (white bar) and total viral DNA (VS and CS strands) (black bar). Data represent the average of three technical qPCR replicates.

Next, we carried out a reconstruction experiment in order to assess the absolute quantification accuracy for each type of TYLCSV ssDNA molecule in the presence of similar amounts of TYLCSV dsDNA. Approximately 10^6^ copies of circular ssDNA VS strand molecules were mixed with equal quantities of circular ssDNA CS strand molecules and dsDNA phagemids (pBTSvs). We then performed a series of assays on this mixture using different primer combinations, as shown in Figure 4. Firstly, we measured the total amount of circular VS molecules following VS-strand DNA synthesis (OCS-TAG primer) and qPCR (OVS and TAG primers). We detected approximately 1.6 × 10^6^ VS molecules, which corresponds to the real amount of VS strands added as ssDNA or dsDNA molecules. Similarly, CS-specific (OVS-TAG primer) first strand synthesis followed by CS-specific qPCR (OCS and TAG primers) detected around 1.4 × 10^6^ CS molecules. When the total number of viral molecules was measured (qPCR using OVS and OCS primers without T4 primer extension), we detected about 3.4 × 10^6^ ssDNA molecules, which roughly corresponds to the total number of VS and CS sequences present in the sample. These results indicate that our method is able to accurately discriminate and quantify each type of molecule within a mixture.

To further test the accuracy of our protocol we performed additional assays with alternative first-strand synthesis and primer combinations. When qPCR with OVS and OCS primers was performed after T4 OCS-TAG extension, 4.8 × 10^6^ molecules of ssDNA were detected. This corresponds to the total amount of circular VS and CS sequences in ssDNA and dsDNA forms detected by standard qPCR (3.4 × 10^6^) plus the additional VS molecules synthesised by T4 DNA polymerase (1.6 × 10^6^). Similarly when CS-strand specific (OVS-TAG) T4 extension was carried out followed by standard qPCR with OVS and OCS primers, 5 × 10^6^ ssDNA molecules were detected, which approximately accounts for the T4-synthesised CS strand copies (1.4 × 10^6^) plus the ssDNA molecules measured by standard qPCR (3.6 × 10^6^). These results confirm that T4 DNA polymerase produces a single copy of each circular molecule present in the sample. Altogether, our results show that this two-step qPCR procedure is a specific, sensitive and accurate technique for determining the number and polarity of begomovirus ssDNA molecules in the presence of cognate complementary strands and host DNA.

### Monitoring viral and complementary strand DNA accumulation in infected plants

**Figure 5:**
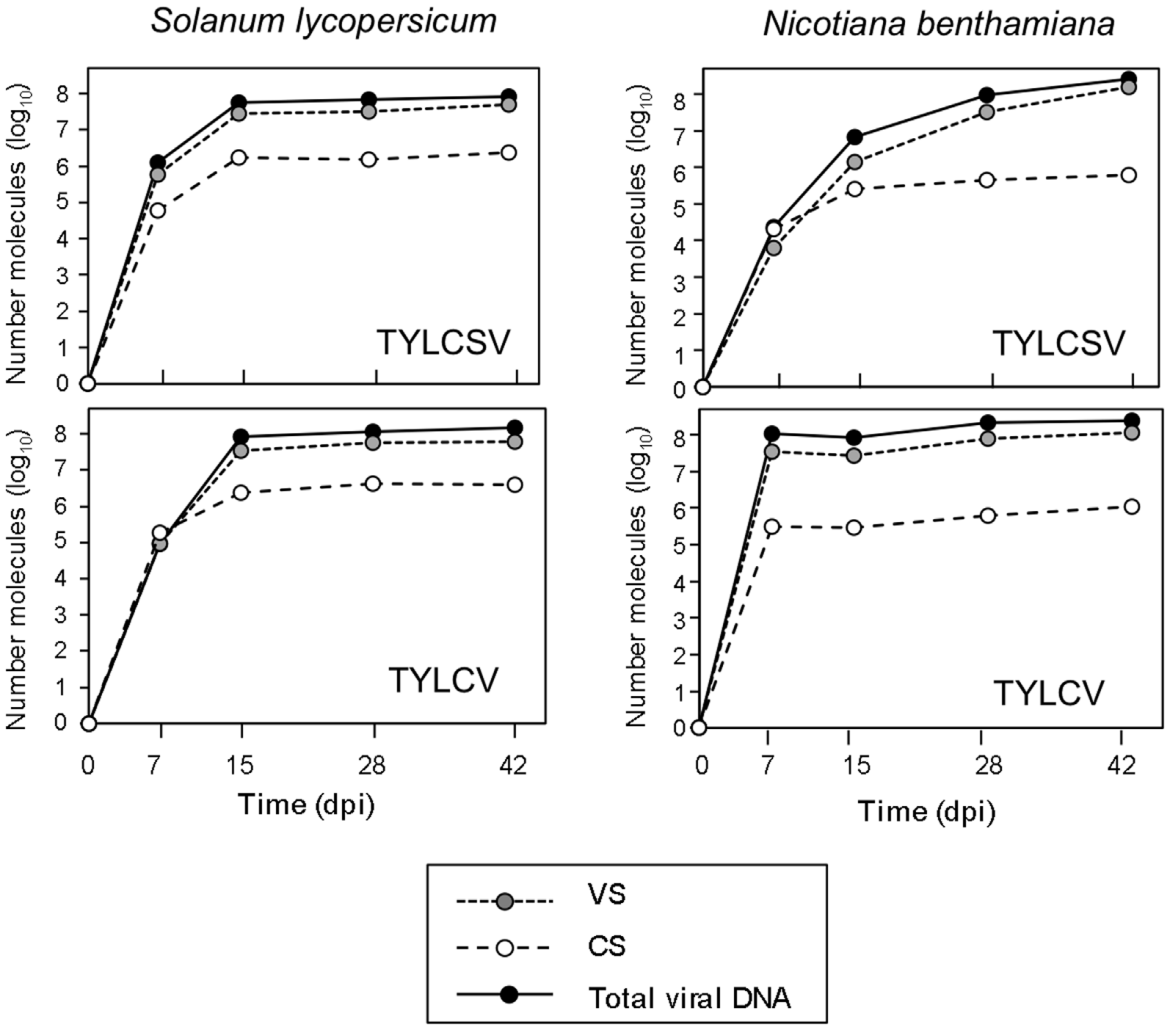
Absolute quantification of virion-sense (VS), or complementary-sense (CS) strands, and total viral DNA of *Tomato yellow leaf curl Sardinia virus* (TYLCSV) and *Tomato yellow leaf curl virus* (TYLCV) during systemic tomato and *N. benthamiana* infections. Plants were inoculated in the axilary bud of the fourth/fifth leaf and two apical leaves from three infected plants were collected at 7, 15, 28 and 42 days post inoculation (dpi). Equal amounts of extracted DNA (1 μg) collected at each time point from the three samples were mixed and 200 ng of the mixture analysed by two-step qPCR to quantify VS or CS strands. Total viral DNA was quantified by standard qPCR.

Next, we used the two-step qPCR procedure to monitor VS and CS strand accumulation during TYLCVS and TYLCV host plant infections. To this end, tomato and *N. benthamiana* plants were inoculated with either TYLCSV or TYLCV and analysed at 7, 15, 28 and 42 days post-inoculation (dpi) (Figure 5). In all cases, the general pattern of viral infection was similar, with the total amount of CS and VS molecules increasing exponentially before reaching a plateau of between 5 × 10^7^ and 1 × 10^8^ molecules per 200 ng of extracted DNA. However, the individual kinetics of viral DNA accumulation varied significantly between viruses and host plants under the experimental conditions used. In tomato, levels of both TYLCSV and TYLCV increased until 15 dpi (Figure 5, left panels). In *N. benthamiana*, the kinetics of the two viruses were highly divergent, with TYLCV and TYLCSV infections reaching maximum levels after 7 and 42 dpi, respectively (Figure 5, right panels).

When we looked at the relative accumulation of individual VS and CS strands, we found that their levels were generally similar during the earlier stages of infection (7 dpi), with the exception of *N. benthamiana* plants infected with TYLCV, where infection progressed much faster (Figure 5). During the later stages of infection, VS strands accumulated much faster than CS molecules. For example at 42 dpi, VS:CS ratios of 20:1 (TYLCSV) or 15:1 (TYLCV) were observed in tomato plants, whereas in *N. benthamiana* plants this ratio was higher for both viruses (254:1 for TYLCSV and 107:1 for TYLCV). The total amount of viral molecules detected at this point of the infection was also over 5 times higher in *N. benthamiana* (1.6 × 10^8^ and 1.2 × 10^8^ for TYLCSV and TYLCV, respectively) than in tomato (4.9 × 10^7^ and 5.9 × 10^7^ for TYLCSV and TYLCV, respectively). Interestingly, when we compared the amounts of VS or CS strands accumulated in tomato and *N. benthamiana* plants at 42 dpi, we noticed that whereas *N. benthamiana* accumulated more VS molecules than tomato (3.1 and 2.0 times for TYLCSV and TYLCV, respectively), the amount of CS strands was higher in tomato than in *N. benthamiana* (3.7 and 3.9 times for TYLCSV and TYLCV, respectively).

To determine the percentage of CS molecules present as dsDNA replicative intermediates or as ssDNA forms during the infection process, the number of CS molecules were quantified and compared with or without denaturing the sample prior to T4 DNA polymerase extension. In the latter case, only ssDNA molecules could be used as template by T4 DNA polymerase and thus be quantified. We analysed samples collected at 7 or 42 dpi from tomato plants infected with TYLCSV or TYLCV. As previously shown (Figure 5), we detected CS molecules in all cases when samples were denatured. However, when no denaturing step was included less than 1% of CS molecules were detected (Table 1). This result indicates that more than 99% of CS molecules were present in the plant as part of dsDNA replicative intermediates.

**Figure 6:**
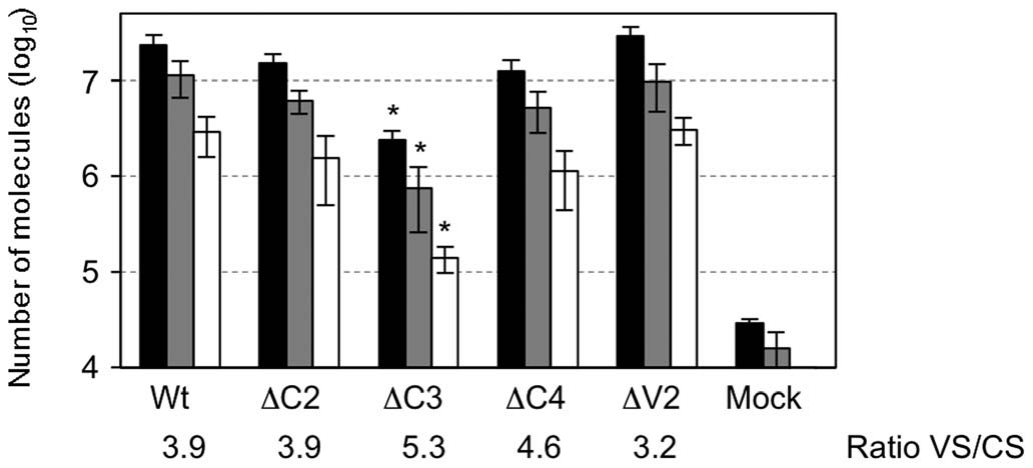
Absolute quantification of virion-sense (VS), or complementary sense (CS) strands, and total viral DNA in local infections of *Tomato yellow leaf curl Sardinia virus* (TYLCSV) in *Nicotiana benthamiana* leaves agroinfiltrated with either the wild type virus or single *C2*, *C3*, *C4* and *V2* TYLCSV mutants. For each combination two leaves from each of three plants were infiltrated with an *Agrobacterium tumefaciens* culture containing the corresponding infectious clone. Mock plants were infiltrated with bacterial cultures harbouring a binary empty vector. DNA was extracted from inoculated leaves of each plant 3 days post infiltration, treated with DpnI and analysed by two-step qPCR to quantify VS or CS strands. Total viral DNA was quantified by standard qPCR. As a control for DpnI digestion, 10^9^ copies of a dsDNA plasmid containing a copy of the TYLCSV genome were added to the mock samples prior to restriction enzyme treatment. Values correspond to the average levels of VS strands (grey bar), CS strands (white bar) and total viral DNA (VS and CS strands) (black bars) from three different plants. Average VS and CS strand ratios obtained from three independent biological replicates are shown. Error bars represent SD. Asterisks indicate a statistically significant difference according to Student's t-test (P < 0.05).

Finally, we used the two-step quantification procedure to determine whether the absence of any of the four proteins that are not essential for viral replication (C2, C3, C4 or V2) affect the accumulation of CS or VS strands. To this end, replication assays were performed on *N. benthamiana* leaves by agroinfiltration of binary plasmids containing infectious wild type or *C2*, *C3*, *C4* or *V2* mutant TYLCSV clones. Quantification of VS and CS molecules as well as the total amount of viral DNA using standard qPCR 3 days post-inoculation showed that C2, C4 and V2 mutants did not significantly affect the accumulation of TYLCSV VS and CS molecules when compared to the wild type (Figure 6). However, for the C3 mutant, we observed 15- and 20-fold decreases in VS and CS strand accumulation, respectively. Interestingly, no significant changes in the VS:CS strand ratio were observed in any of the mutants assayed with respect to the wild type TYLCSV (according to Student's t-test, P < 0.05).

## Discussion

The characterisation of viral accumulation during the infection process is essential for understanding virus-host interaction mechanisms. Although the qPCR techniques available to determine accumulation of ssDNA viruses have greatly improved in recent years, they do not discriminate between the type (VS or CS) of viral molecules present. Therefore, the method presented here represents an important step forward in our ability to analyse the infection process of circular ssDNA viruses, since it allows the amount of each of the two strands generated during viral infection to be determined. The novelty lies in the first step that uses two strand-specific primers and the T4 DNA polymerase, which lacks strand displacement activity under the experimental conditions used. As a result, absolute quantification of VS and CS strands can be achieved if the whole process is done in parallel with the corresponding ssDNA standard. We have demonstrated the accuracy and sensitivity of the method over a wide range of viral concentrations for two different geminiviruses (TYLCSV and TYLCV). Moreover, we find that neither sensitivity nor precision were altered by the presence of plant genome DNA or cognate dsDNA.

We tested the potential of this new procedure for studying viral replication mechanisms by monitoring infections of two begomovirus species. Using our two-step protocol to analyse tomato and *N. benthamiana* plants inoculated with TYLCSV or TYLCV we confirmed the dynamics of viral accumulation previously described^25^. Moreover, the method provided novel information about viral replication dynamics. Quantification of VS and CS molecules showed that similar levels of the two strands accumulate during the first phase of the infection process. Considering that CS molecules accumulate almost exclusively as components of dsDNA replicative intermediates (Table 1), our findings suggest that during the initial steps of infection, viral replication takes place mainly through the formation of dsDNA. Once the amount of CS molecules accumulated in the plant reaches a certain level, it seems that the virus alters the replication mechanism toward the generation of ssDNA VS molecules. During this phase of infection the quantity of CS molecules does not vary, while the amount of VS molecules increases exponentially. It is likely that these VS molecules are destined to be encapsidated to form virions and complete the propagation cycle of the virus. Finally, we observed that viral accumulation eventually slows down and reaches a plateau that is then maintained. Our results show that the time frame over which these viral replication changes occur is dependent on plant host and viral species when cultured under the same growth conditions. Despite wide variations in timing, all the infections monitored exhibited the same overall pattern of viral replication/accumulation, suggesting that these dynamics might be a common feature of geminivirus infection.

The strand-specific quantification procedure described here allowed us to dissect and compare the infection process of different begomoviruses. Thus, in spite of the general similarity between VS/CS accumulation patterns, detailed analysis of the data revealed some interesting differences between viruses and host-plants. Although the amount of TYLCV or TYLCSV VS molecules accumulated at the end of the infection was lower in tomato than in *N. benthamiana*, the amount of CS molecules was higher in the former host. This might indicate that a larger number of dsDNA replicative forms are required to infect tomato plants. The efficiency of generating VS ssDNA from dsDNA intermediates was 10 times lower in tomato than in *N. benthamiana*. Reduced expression of viral genes, or a lower hijacking efficiency of the host machinery in tomato, might explain the differences observed between the two plant species.

The quantification procedure presented here was also used to determine the need of specific begomovirus proteins for viral strand accumulation. We demonstrate that null mutations in three TYLCSV genes (*C2, C4* and *V2*) did not abolish viral replication nor significantly modify the VS to CS ratio detected in tissues infiltrated with wild type virus. This confirms previous observations^26^,^27^ indicating that the mutation of *C2*, *C4* or *V2* does not affect viral replication. However, here we extend those results by showing that mutation of the *C3* gene encoding the putative replication enhancer protein resulted in reduced accumulation of both types of viral molecules by approximately 20 times with respect to the wild type. Although reduced viral accumulation in C3 mutant infections has already been described for other geminiviruses^28^,^29^,^30^,^31^,^32^, this is the first time that this decrease has been shown to affect the synthesis of both strands. These findings indicate that C3 is involved in the generation of both ds and ssDNA molecules.

The quantification technique implemented here has the potential to shed light on several other open questions about ssDNA virus replication. For example, as to whether begomovirus replication takes place in the whitefly *Bemisia tabaci* insect vector, and if so, whether viral replication is dependent on the whitefly genotype. Such issues are pivotal for understanding viral epidemics. It is also worth mentioning that although in this work we have focused on demonstrating the feasibility of the proposed technique for quantifying VS and CS strands generated during begomovirus infection, the method could easily be adapted to characterise the replication and accumulation of plasmids or any virus with a DNA circular genome by using appropriate specific primers.

## Methods

### Microorganisms and general methods

*Escherichia coli* strains and nucleic acids were manipulated according to standard methods^33^. *E. coli* strain DH5-α was used for subcloning and construct storage. *E. coli* strain XL1 BLUE F′ and the M13KO7 helper phage were used for production of geminivirus-derived single-stranded DNA standards for qPCR. *Agrobacterium tumefaciens* strain GV3101 was used for transient and systemic infection assays using infectious begomovirus clones.

### Infectious viral clones

Infectious clones of wild type ES TYLCSV strain (pGTYA14) and Mild TYLCV strain (p1.7SP72/97) isolates have been described previously by^34^ and^35^, respectively. Generation of TYLCSV C2_T2C_ (ΔC2), C4_T2C_ (ΔC4), V2_T2G_ (ΔV2) mutant clones are described elsewhere^34^,^36^. The TYLCSV ΔC3 mutant (containing a 72 bp deletion) was generated by two-sided splicing overlap extension^37^ using pGTYA14 as template and two primer pairs for the two initial PCRs: ΔC31F/ΔC31R (5′-CGAAAGCCGCGGATGTAC-3′/5′-AATGAGGCTAGCGTTACCTTGAGAACACAATAGATG-3′) and ΔC32F/ΔC32R (5′-GGTAACGCTAGCCTGATTCTTATACACTTGAAACCTAAAG-3′/5′-GGAGGCATACCCACTATC-3′). Subsequent amplification used primer pair ΔC31F/ΔC32R. A PCR fragment containing the *C3* deletion was cloned into the pGEM-T Easy vector (Promega) using TA cloning technology according to the manufacturer's instructions to obtain pGEM-ΔC3. An infectious clone was generated by replacing the SacI/NcoI fragment containing the wild type pGTYA14 *C3* with the mutant pGEM-ΔC3 version to yield pGTSΔC3.

### Primer design

Viral strand-specific primers for T4 DNA polymerase DNA synthesis containing a 5′ Tag sequence were OCS-TAG (5′-AGTTTAAGAACCCTTCCCGCGGACTTTACATGGGCCTTCAC-3′) for VS determination and OVS-TAG (5′-AGTTTAAGAACCCTTCCCGCGAAGGCTGAACTTCGACAGC-3′) for CS determination.

Strand-specific qPCR primers OCS (5′-GGACTTTACATGGGCCTTCAC-3′) and OVS (5′-GAAGGCTGAACTTCGACAGC-3′) were designed against conserved regions of old-word begomovirus CP ORFs using the Primer Blast tool (http://www.ncbi.nlm.nih.gov/tools/primer-blast/). For a nucleotide alignment of primers see Supplementary Fig. S2. Strand-specific primers were combined with the TAG oligonucleotide (5′-AGTTTAAGAACCCTTCCCGC-3′) for qPCR assays. The tomato 25S nuclear ribosomal RNA gene (25S rRNA) (GenBank Accession no. X13557), which is highly conserved in *Solanaceae*, was used as a reference for qPCR normalisation, with amplification performed using primers 25S UNIV (+) (5′-ATAACCGCATCAGGTCTCCA-3′) and 25S UNIV (−) (5′-CCGAAGTTACGGATCCATTT-3′)^18^.

### Synthesis of single-stranded DNA standards

A 2.7 kb EcoRI fragment of pGTYA14 containing the full length TYLCSV genome was cloned into the pBSK II (−) EcoRI site. The orientation of the insert relative to the f1 phage replication origin was determined by SalI digestion to select phagemids that produce VS (pBTSvs) or CS (pBTScs) ssDNA.

SsDNA and dsDNA TYLCV standards were also generated. For this, rolling circle amplification (RCA) with φ29 DNA polymerase was performed on total DNA extracted from *N. benthamiana* infected with TYLCV isolate SP72/97. RCA product was digested with BamHI to obtain a 2.7 kb fragment representing a full-length copy of the viral genome and cloned into the BamHI site of pBSK II (−). The orientation of the insert relative to the f1 phage replication origin was determined by SalI digestion to select phagemids that produce VS (pBTYMvs) or CS (pBTYMcs) ssDNA.

To obtain single-stranded circular molecules, *E. coli* XL1 BLUE bacteria were first transformed with pBTSvs, pBTScs, pBTYMvs or pBTYMcs and then infected with a helper virus. Briefly, 1.5 ml of TYP media (1.6% w/v Bacto-triptone, 1.6% w/v yeast extract, 8.5 mM NaCl, 1 mM K_2_HPO_4_) containing 100 μg/ml ampicillin was inoculated with a fresh colony and 2 μl of a M13 helper virus suspension (final concentration 10^7^ pfu/ml), and grown with vigorous shaking at 37°C for 60 minutes. Kanamycin was then added to a final concentration of 70 μg/ml and grown for a further 14–18 h. Each culture was centrifuged at 13,000 rpm for 10 minutes, its supernatant transferred to a new tube, 200 μl of PEG solution (20% w/v PEG 8000, 2.5 M NaCl) added and mixed gently. The resulting mixture was centrifuged at 13,000 rpm for 10 minutes, and the pellet resuspended in 100 μl of TE. Phagemid solutions were then treated twice with phenol/chloroform and precipitated with NaOAc and absolute ethanol, and resuspended in 20 μl of water. Single-stranded circular molecules containing virus sense (ssVS) and complementary sense (ssCS) TYLCSV, and virus sense (ssVS-TYM) and complementary sense (ssCS-TYM) TYLCV-Mild, were analysed by gel electrophoresis and with a NanoDrop 8000 spectrophotometer (ThermoFisher Scientific, Courtaboeuf, France) to assess purity (according to A260/A280) and yield.

### Absolute quantification of VS and CS strands by two-step qPCR

Quantification of VS and CS molecules was performed by two-step qPCR as shown in Figure 1. T4 DNA polymerase (TAKARA, Shiga, Japan) was used for the extension reaction. Reactions were performed in 10 μl containing 1 μM primer, 1% BSA and 1 μM dNTPs in T4 DNA polymerase buffer. Reaction mixes were denatured (except where indicated) at 95°C for 10 minutes, cooled down to room temperature, and incubated at 37°C for 30 minutes with 1 unit of T4 DNA polymerase. Following the reaction, primers were removed using the QIAquick purification kit (QIAGEN, Hamburg, Germany) according to the manufacturer's instructions, and DNA eluted in 50 μl of water.

For the second step, 2 μl of the first-strand reaction were mixed with 18 μl of SsoFast EvaGreen Supermix (Bio-Rad, Hercules, USA) (10 μl EvaGreen supermix, 1 μl of each specific primer 10 μM, 6 μl of nuclease free water) following manufacturer's instructions, and subjected to one cycle at 95°C for 30 s, forty cycles at 95°C for 10 s and 60°C for 15 s in a MyiQ real time cycler (Bio-Rad, Hercules, USA), using the appropriate primer pair combination. Melting curves were obtained after a final step of 60 cycles under a ramp rate of 0.5°C every 10 seconds from 60 to 90°C.

To create standard curves for the absolute quantification of VS and CS, serial dilutions containing between 10^7^ and 10^3^ molecules of circular ssDNA or dsDNA per μl were used. Standard curves were obtained by linear regression analysis of the quantification cycle (C_q_) value of each of the three technical replicates over the log_10_ of the amount of DNA. qPCR efficiency (E) was calculated as follows: E = e^(ln10/−s)^ − 1 where a slope s = −3.322 represents an efficiency of 100%. Quantification of both VS and CS strands was obtained by extrapolation of C_q_ data with the corresponding standard curve. Data from dsDNA quantification was multiplied by two since each dsDNA molecule contains two ssDNAs (VS + CS). To normalise qPCR data, the 25S rRNA amplicon was amplified from each sample. The resulting C_q_ values allowed the relative differences between samples to be calculated according to the 2^−**ΔCt**^ formula and then used to generate normalised data. To quantify dsDNA molecules in viral infection assays, 2 μl of DNA extract was used, whereas for ssDNA quantification, 2 μl of the T4 DNA polymerase reaction was used.

### Systemic and local geminivirus infections

For systemic infections, *N. benthamiana* and tomato plants were agroinoculated with TYLCSV and TYLCV infectious clones in the axillary bud of the fourth/fifth leaf of 3 to 4 week-old plants. For local infection assays in *N. benthamiana*, two fully expanded leaves of six week-old plants were infiltrated with *A. tumefaciens* containing infectious clones of TYLCSV; or ΔC2, ΔC3, ΔC4 or ΔV2 TYLCSV mutant clones. After harvesting, samples were immediately frozen and ground in liquid nitrogen and stored at −80°C until processing.

Total DNA was extracted from 100 mg of young uninoculated tissues using a CTAB-based method^38^ at different times post-inoculation or from the infiltrated areas 3 days post inoculation (dpi). Purity (according to A260/A280 ratio) and yield were assessed with a NanoDrop 8000 spectrophotometer (ThermoFisher Scientific, Courtaboeuf, France). For transitory assays 1 μg of total DNA was digested with DpnI to eliminate prokaryotic DNA input. Samples were heated (80°C for 10 minutes) to inactivate the restriction enzyme, and 200 ng of DNA used for quantification of VS or CS molecules. As a control to assess DpnI endonuclease efficiency, 10^9^ molecules of pBTSvs (phagemid containing the complete TYLCSV genome) were added to 1 μg of DNA extracted from mock-inoculated plants and treated in parallel.

## Supplementary Material

Supplementary Information Supplementary information

## Figures and Tables

**Table 1:** Absolute quantification of CS strands present in the form of dsDNA molecules in tomato plants infected with *Tomato yellow leaf curl Sardinia virus* (TYLCSV) or *Tomato yellow leaf curl virus* (TYLCV). Two to three apical leaves from three infected plants were collected at 7 and 42 days post inoculation (dpi). An equal amount of extracted DNA (1 μg) from the three samples collected at each time point were mixed. CS molecules present in these DNA samples were quantified using the two-step procedure, with (denaturing) or without heating (non-denaturing) the samples prior to T4 DNA polymerase extension reaction. Percentages of CS strands present as part of dsDNA molecules were calculated based on the proportion of CS strands detected under non-denaturing conditions relative to their amount under denaturing conditions

	TYLCSV		TYLCV	
	7 dpi	42 dpi	7 dpi	42 dpi
*Non-denaturing*	Nd^a^	1.3 × 10^4^	Nd	5.1 × 10^4^
*Denaturing*	1.2 × 10^5^	3.0 × 10^6^	1.1 × 10^5^	6.1 × 10^6^
*% dsDNA*	100.0	99.6	100.0	99.2

^a^Nd: CS molecules below the level of qPCR detection.
