# Supplementary material for: A sensitive method for the quantification of virion-sense and complementary-sense DNA strands of circular single-stranded DNA viruses
Source: Sci Rep. 2014 Sep 22;4:6438. doi: 10.1038/srep06438 (PMC5377365; doi:10.1038/srep06438)
Supplement: Supplementary Information [file srep06438-s1.pdf]

Supplementary information

A sensitive method for the quantification of virion-sense and complementary-sense DNA strands of circular single-stranded DNA viruses

Edgar A. Rodríguez-Negrete[1], Sonia Sánchez-Campos[2], M. Carmen Cañizares[2], Jesús Navas-Castillo[2], Enrique Moriones[2], Eduardo R. Bejarano[1] and Ana Grande-Pérez[1, *]

[1] Instituto de Hortofruticultura Subtropical y Mediterránea “La Mayora” (IHSM-UMA-CSIC), Universidad de Málaga - Consejo Superior de Investigaciones Científicas, Área de Genética, Campus de Teatinos, 29071 Málaga, Spain
[2] Instituto de Hortofruticultura Subtropical y Mediterránea "La Mayora" (IHSM-UMA-CSIC), Universidad de Málaga - Consejo Superior de Investigaciones Científicas, Estación Experimental "La Mayora", 29750 Algarrobo-Costa, Málaga, Spain.

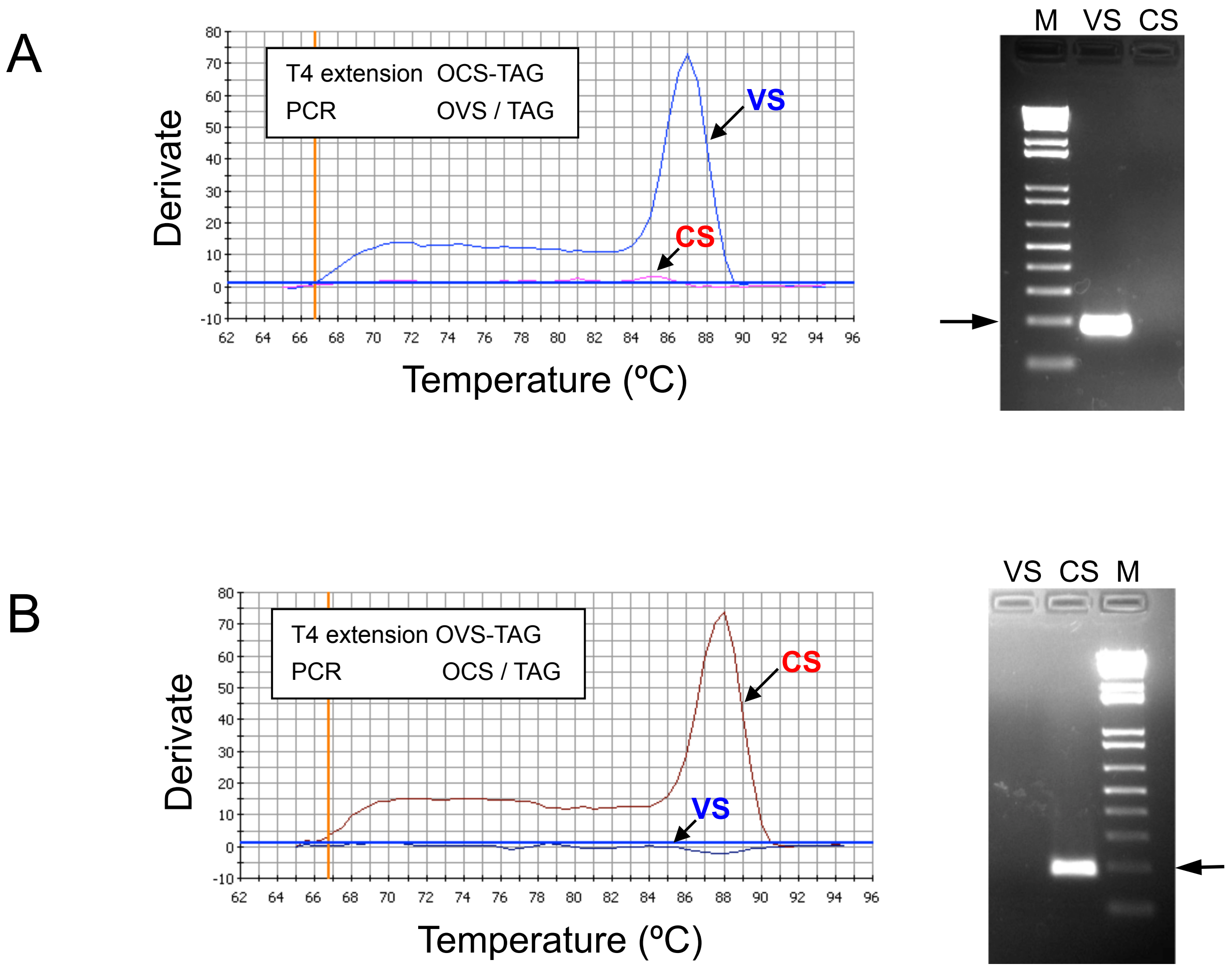

Supplementary Figure S1. Specificity of the two-step qPCR procedure. Virion-sense (VS) or complementary-sense (CS) circular single-stranded DNA molecules from *Tomato yellow leaf curl Sardinia virus* (TYLCSV) were used to evaluate the specificity of the two-step qPCR procedure. For detection of VS strands (A), samples containing 10^7 molecules of circular ssDNA bearing the VS strand and 10^8 molecules of circular ssDNA bearing the CS strand were used. For detection of CS (B) samples containing 10^7 molecules of circular ssDNA bearing the CS strand and 10^8 molecules of circular ssDNA bearing the VS strand were employed. Products from T4 DNA polymerase reactions using first strand synthesis primers OCS-TAG (A) or OVS-TAG (B) were amplified by qPCR using the OVS and TAG primers (for VS) or OCS and TAG primers (for CS). The corresponding qPCR amplification melting curves are shown (left). qPCR products were also separated by electrophoresis on a 2% agarose gel (right). M: 1 kb molecular ladder. Arrow indicates a 200 bp band.

**ACMV-[NG]**	(X17095)	503	GAAGGCTGAACTTCGACAGC	523
**EACV-[CM]**	(AF112354)	397	GGAGGCTGAACTTCGACAGC	417
**PYVMV**	(Y502935)	354	GAAGGCTGAACTTCGACAGC	374
**ToLCCNV**	(AJ558119)	341	GTCGGCTGAACTTCGACAGC	361
**TYLCV-[Alm]**	(AJ489258)	370	GAAGGCTGAACTTCGACAGC	390
**TYLCV-Mld**	(X76319)	532	GAAGGCTGAACTTCGACAGC	552
**TYLCSV-[ES2]**	(L27708)	364	GAAAACTGAACTTCGACAGC	384
		**Primer OVS**	**5´-GAAGGCTGAACTTCGACAGC-3´**	

**ACMV-[NG]**	(X17095)	653	GTGAAGGCCCATGTAAGGTCC	673
**EACV-[CM]**	(AF112354)	544	GTGAAGGCCCATGTAAGGTTC	564
**PYVMV**	(Y502935)	504	GTGAGGGTCCGTGTAAGGTTC	524
**ToLCCNV**	(AJ558119)	490	GTGAGGGTCCATGTAAGGTCC	510
**TYLCV-[Alm]**	(AJ489258)	520	GTGAAGGCCCATGTAAAGTCC	530
**TYLCV-Mld**	(X76319)	682	GTGAAGGCCCATGTAAAGTCC	702
**TYLCSV-[ES2]**	(L27708)	511	GTGAAGGTCCTTGTAAAGTCC	531
		**Primer OVS**	**3´-CACTTCCGGGTACATTTCAGG-5´**	

Supplementary Figure S2. Nucleotide alignment of conserved coat protein-encoding gene regions selected for primer design, composed of *African cassava mosaic virus*-[Nigeria] (ACMV-[NG]); *East African cassava mosaic virus*-[Cameroon] (EACMV-[CM]); *Pepper yellow vein Mali virus* (PYMV); *Tomato leaf curl China virus* (ToLCCNV); *Tomato yellow leaf curl virus*-Mild; (TYLCV-Mld); *Tomato yellow leaf curl Sardinia virus*-Spain (TYLCSV-ES) (virus genome amplicon location and GenBank accession numbers are indicated); primers OCS and OVS are shown at the bottom in bold letters; nucleotide mismatches with primer sequences are indicated in red
